# PARP1 as a novel therapeutic and diagnostic tool in autoimmune rheumatic diseases: a systematic literature review

**DOI:** 10.1007/s00296-026-06187-0

**Published:** 2026-06-09

**Authors:** Malini Dey, Mrinalini Dey

**Affiliations:** 1 Autism Research Centre, Department of Psychiatry, University of Cambridge, Douglas House, 18b Trumpington Road, Cambridge, CB2 8AH UK; 2 Centre for Rheumatic Diseases, Weston Education Centre, King’s College London, Cutcombe Road, London, SE5 9RJ UK

**Keywords:** PARP1, PARP inhibitor, Autoimmune Rheumatic Diseases, Translational science, Polymorphisms, Biomarker

## Abstract

ADP-ribosylation is a reversible post-translational modification regulated by poly(ADP-ribose) polymerases (PARPs), a family of enzymes involved in DNA repair, transcriptional regulation, and immune responses. Among the 17 known PARP family members, PARP1 is the most extensively studied in autoimmune rheumatic diseases (ARDs). Although increasing evidence implicates PARP1 in ARD pathogenesis, its potential diagnostic and therapeutic relevance has not been systematically synthesised. This systematic literature review (SLR) aimed to evaluate the role of PARP1 in the pathogenesis, diagnosis and treatment of ARDs. MEDLINE and Embase databases were searched on 6th May 2026 using MeSH keywords for systemic ARDs, PARP1 and PARP inhibitors. Studies involving adult ARD patients and relevant animal models were included. Of 3564 records identified (post-deduplication), 41 studies were included. Seventeen studies included human ARD cohorts and nine used murine models recapitulating specific ARDs. Most studies focused on systemic lupus erythematosus (SLE; *n* = 18), rheumatoid arthritis (*n* = 14), and systemic sclerosis (SSc; *n* = 7). Across experimental models, inhibition or genetic disruption of PARP1 frequently attenuated inflammatory responses, reducing pro-inflammatory mediator expression, oxidative stress, and tissue damage. However, PARP1 also demonstrated context-dependent regulatory effects in immune signalling pathways. In SLE and SSc, reduced PARP1 activity and impaired DNA repair capacity were reported. Genetic association studies produced heterogeneous findings, while several studies identified PARP1-related autoantibodies or activity changes as potential diagnostic biomarkers, particularly in SLE. Overall, our SLR highlights PARP1 as an important regulator of immune pathways in ARDs, with potential relevance for future diagnostic and therapeutic strategies. PROSPERO: CRD420251000954.

**Supplementary Information:**

The online version contains supplementary material available at 10.1007/s00296-026-06187-0.

## Introduction

ADP-ribosylation is an evolutionary-conserved post-translational modification, catalysed by a class of ADP-ribosyltransferases (ARTs) called poly(ADP-ribose) polymerases (PARPs) [1, 2]. ADP-ribosylation governs multiple cellular processes including DNA repair, inflammation and immune cell development, and is therefore implicated across numerous diseases such as autoimmune rheumatic diseases (ARDs) [3, 4]. ARDs comprise a heterogeneous group of chronic immune-mediated disorders, characterised by a loss of immune tolerance and persistent inflammation affecting the joints, connective tissues, and multiple organ systems [5, 6]. Examples include rheumatoid arthritis (RA), systemic lupus erythematosus (SLE) and systemic sclerosis (SSc) [7–9]. ARDs arise due to complex interactions between genetic susceptibility, environmental triggers and dysregulated innate and adaptive immune responses [5]. Despite advances in immunomodulatory therapies, ARDs remain associated with substantial morbidity, organ damage and impaired quality of life, highlighting the need to better understand the molecular mechanisms driving disease initiation and progression [10–12].

To date, there are 17 known members of the PARP enzyme family [13, 14]. PARPs regulate immunobiological processes including pro-inflammatory gene transcription and expression, stimulation of pro-inflammatory signal transduction pathways, differentiation and activation of innate and adaptive immune cells, and antibody production [15, 16]. PARP1 accounts for ~ 90% cellular ADP-ribosylation activity [17, 18] and is activated by oxidative stress and pro-inflammatory mediators. Genomic instability, arising due to inflammatory DNA damage and reparatory mechanisms, can lead to PARP1 over-activation due to extreme depletion of NAD^+^ and ATP, further exacerbating inflammation and cell death [19, 20].

PARP enzymatic activity is vital for maintaining the balance between pro-inflammatory and anti-inflammatory responses. Pharmacological inhibition of PARP1 has conferred protective roles in experimental models recapitulating ARDs, by rendering anti-inflammatory effects, such as downregulating pro-inflammatory mediator expression, disrupting the recruitment and migration of immune cells, attenuating dysfunctionalities associated with oxidative DNA damage, and enhancing anti-inflammatory mediator production [21, 22]. Previous studies have explored specific aspects of PARP-related biology in ARDs, including PARP1 polymorphisms in RA and apoptosis-associated PARP signalling within RA fibroblast-like synoviocytes [23–26]. However, no previous review has systematically synthesised the broader evidence relating to PARP 1 across ARDs, including its roles in pathogenesis, biomarker development and therapeutic targeting.

This systematic literature aims to explore the role of PARP1 in the (i) pathogenesis and diagnosis, and (ii) treatment, of ARDs. The ultimate aim is to highlight the potential application of PARP1 both as a potential therapeutic and diagnostic tool for ARDs.

## Methods

This SLR was conducted in accordance with the Cochrane Handbook and reported as per the Preferred Reporting Items for Systematic Reviews and Meta-Analyses guidelines [27, 28]. The protocol was registered on PROSPERO (CRD420251000954).

### Data sources and searches

To ensure a broad capture of relevant studies, adults (≥ 18 years) with a confirmed ARD diagnosis, as well as animal studies, were included. Studies in paediatric populations and in people with non-immune-mediated rheumatic diseases were excluded. Studies were required to assess the role(s) of PARP, and inhibitors of PARP, in the pathogenesis and as potential targets for ARD treatment.

A comprehensive search strategy was developed and performed on 6th May 2026 (Supplementary Material 1). MeSH headings and keywords were searched within Medline and Embase, with no time restriction.

### Study selection

Both randomised and non-randomised study types were considered, including: observational studies, randomised controlled trials, case series. Non-clinical, laboratory-based and basic science studies were also included but analysed separately. The following studies were excluded: review articles, opinion articles (including editorials), single case reports and case series < 5. Reference lists from relevant systematic reviews were hand-searched. English-language articles only were included.

All full-length articles identified in the search were uploaded into EndNote V.21 (Clarivate Analytics), with duplicates removed. Titles and abstracts of all retrieved articles were screened independently by both authors with disagreements resolved through further discussion.

The main outcomes analysed were: (i) changes in PARP1 expression or activity in ARDs (measured in tissues, cells or serum); (ii) effect of PARP1 modulation (inhibitors, activators, gene knockdown or knockout) on disease activity, inflammation or tissue damage; (iii) impact on innate and adaptive immune cell differentiation, activation and function; and (iv) clinical outcomes in human studies (e.g. disease severity, remission rates, biomarker changes). The additional outcomes analysed were: (i) mechanistic insights from in vitro studies (e.g. inflammatory signalling pathways); (ii) adverse effects of PARP1 treatment and modulation; (iii) differences between experimental models in animal studies; and (iv) biomarker correlations between preclinical and clinical findings.

### Data extraction and synthesis

A study-specific extraction table was used to collate information from each included study on: study design, method of targeting PARP1 (where applicable), type of ARD(s), model system(s) used, total number of participants (where applicable), intervention details, and key outcomes. Data were extracted independently by one author, with 20% validation carried out by the second. Studies including mixed cohorts or non-ARD comparator conditions were retained where PARP1-related findings were considered mechanistically relevant to ARD pathophysiology. In such cases, data extraction focused specifically on findings relevant to immune-mediated inflammatory or fibrotic processes.

Outcomes from all included studies were collated as a narrative synthesis, including a summary of study characteristics, participant demographics (where applicable) and intervention details. Findings were considered based on study design, disease type, and intervention type, as well as the role of PARP1.

## Results

### Search results

**Fig. 1:**
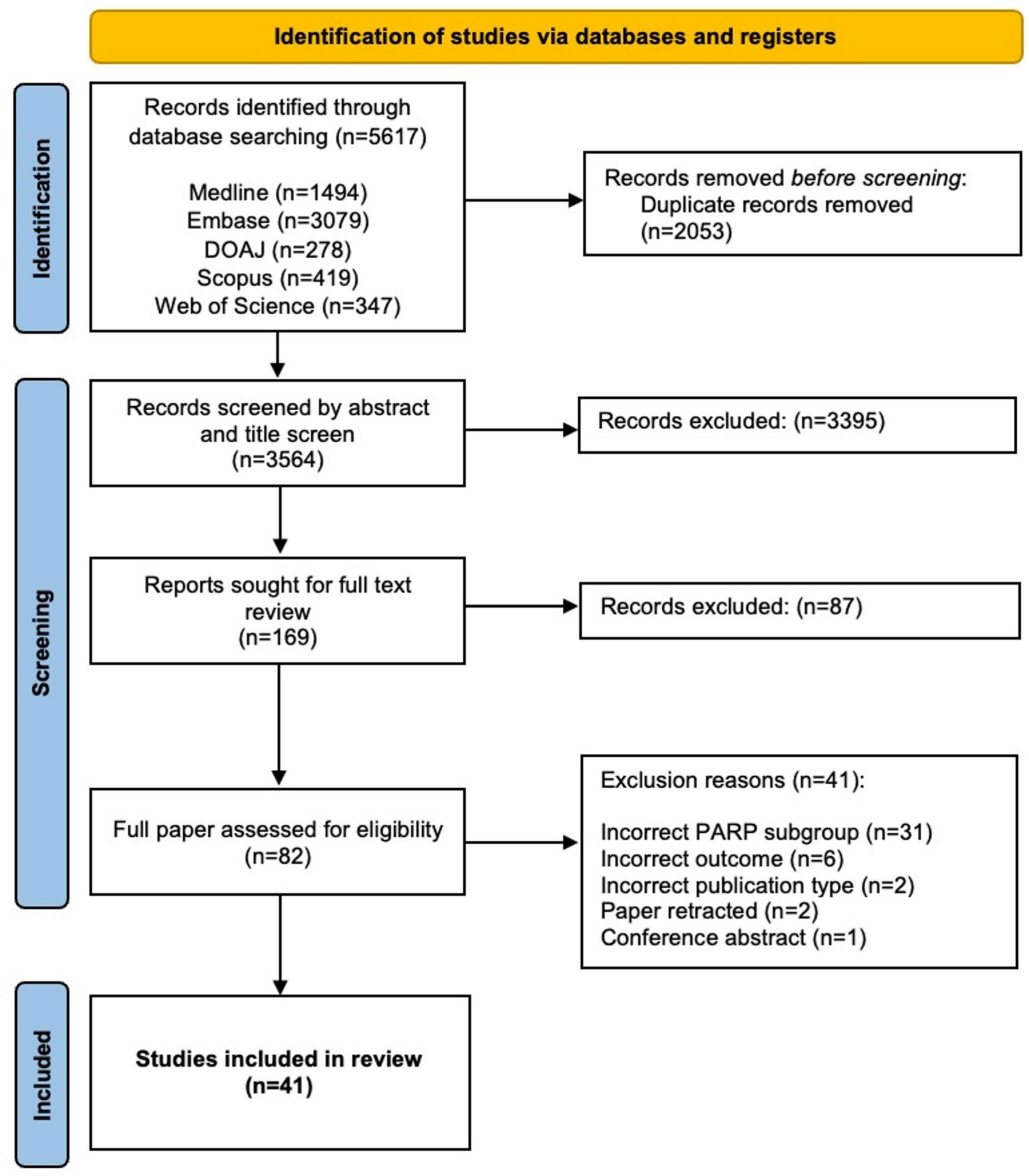
PRISMA flow diagram of included studies

**Fig. 2:**
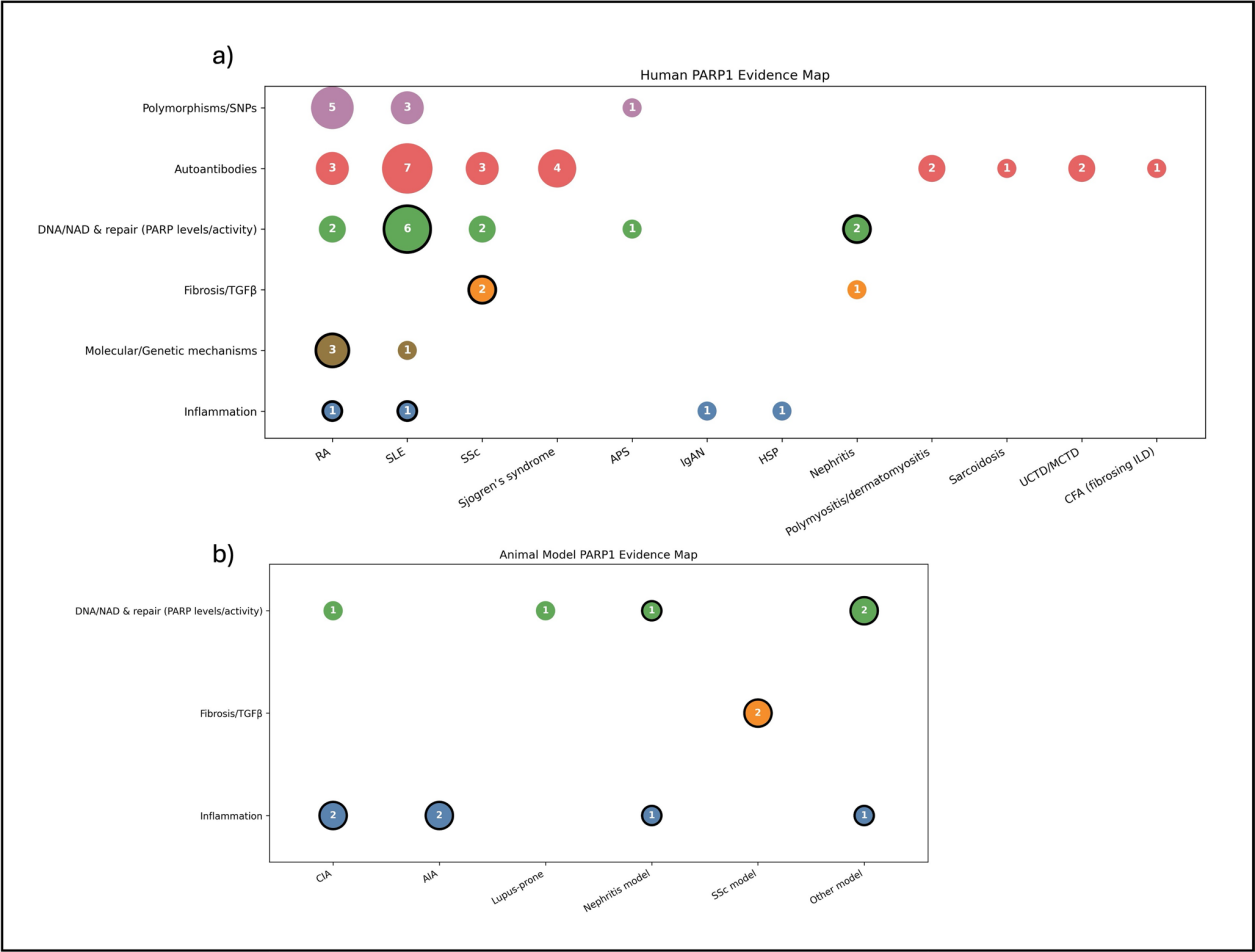
Evidence map of PARP1 research in autoimmune rheumatic diseases.Bubble plots summarise the distribution of the published PARP1 studies across the autoimmune rheumatic disease groups (x-axis) and the biological outcome domains (y-axis) in humans (**a**) and animal models (**b**). Bubble size and colour reflect the number of studies within each disease–domain combination. Circles with a black outline indicate at least one study which tested PARP modulation (e.g. pharmacological inhibition, genetic perturbation or NAD⁺ boosting). Python was used to generate the figures. *PARP* poly(ADP-ribose) polymerase 1; *RA* rheumatoid arthritis; *SLE* systemic lupus erythematosus; *SSc* systemic sclerosis; *APS* antiphospholipid syndrome; *IgAN* IgA nephropathy; *HSP* henoch schonlein purpura; *UCTD* undifferentiated connective tissue disease; *MCTD* mixed connective tissue disease; *CFA* cryptogenic fibrosing alveolitis; *ILD* interstitial lung disease; *CIA* collagen antibody-induced arthritis; *AIA* adjuvant-induced arthritis; *SNP* single nucleotide polymorphism; *DNA* deoxyribonucleic acid; *NAD* nicotinamide adenine dinucleotide; *TGFß* transforming growth factor ß

The initial search retrieved a total of 5617 records (Fig. 1). Following deduplication, 3564 papers were taken forward for screening. Ultimately, 41 articles were included in the SLR. Results are summarised in Supplementary Table 1. With regards to the population under study, 28 studies included ARD patients (Fig. 2a) and 11 employed murine models recapitulating specific ARDs (Fig. 2b). In terms of targeting PARP1, 13 studies used either or a combination of PARP1 siRNA, PARP1 shRNA, PARP1 inhibitor and PARP1 knockout model. Most studies focused on SLE (*n* = 18), RA (*n* = 14) and SSc (*n* = 7).

Due to the types of studies ultimately included (i.e. translational science and animal models), it was not possible to undertake a risk of bias assessment.

### Characteristics of included studies

#### Methods of targeting PARP1

13 studies employed methods of targeting PARP1 using either or a combination of PARP1 siRNA, PARP1 shRNA and PARP1 inhibitor [29–41] out of which one of these studies used mice lacking PARP1 [41]. 12 studies used an inhibitor including: 3-aminobenzamide (3-AB) [29, 32, 36, 37, 40], 5-aminoisoquinolinone (5-AIQ or AIQ) [30, 34, 35], PJ34 [39, 40], nicotinamide [38], benzamide [31], DPQ and 4-amino-1,8-naphthimide (ANI) [33].

### Types of autoimmune rheumatic disease (ARD)

With regards to ARDs, when considering both human and animal studies, 18 articles focussed on SLE [31, 32, 39, 42–56]; 14 on RA [23, 24, 33, 36, 37, 47, 49, 51, 57–62]; seven on SSc [40, 42, 43, 47, 49, 51, 63]; four on primary and secondary Sjogren’s disease (SjD) [47, 49, 51, 55]; three on autoimmune nephritis [35, 46, 52]; two on adjuvant-induced arthritis (AIA) [29, 30]; two on antiphospholipid syndrome (APLS) [44, 48]; two on unspecified arthritis [38, 46]; two on autoimmune myositis (including polymyositis and dermatomyositis) [47, 49]; two on mixed or undifferentiated connective tissue disease [51, 55]; one on lupus nephritis [64]; one on sarcoidosis [51]; one on primary IgA nephropathy (IgAN) and IgA-mediated disorder: Henoch-Schönlein purpura [65]; one on juvenile chronic arthritis [55]; one on SLE-like autoimmune disease [52]; one on generalised systemic autoimmunity [52]; and one on cryptogenic fibrosing alveolitis (CFA) [51].

### Non-ARD conditions

Of note, one study enrolled patients with several types of ARDs, including sarcoidosis, but also patients with CFA [51]. Three studies used murine models with collagen antibody-induced arthritis (CIA), which shares many clinical, histologic and immunologic features with RA [34, 41, 61]. A further study recruited people who have ARDs, but did not specify which type [66].

### Model systems used

17 studies involved ARD patients [23, 24, 31, 39, 40, 42–46, 48, 54, 57, 59, 60, 63, 65] while 10 involved murine models recapitulating specific ARDs [29, 30, 32, 34, 35, 38, 40, 41, 52, 56]. A range of human and murine sample types were obtained from ten studies including sera [47, 49–51, 53, 55, 66], blood [32, 52], synoviocytes [33], synovial tissue [36] and skin biopsies [40]. Of note, four studies used human cell lines [32, 37, 65, 66], one studied RNA from human and murine tissues (using RT-PCR and cDNA preparation) [64] and one study leveraged RNA-seq datasets from people with RA, and mice [58].

### Molecular mechanisms and treatment

- Exacerbated role of PARP1 in ARD pathogenesis

25 papers focused on the role of PARP1 in the molecular mechanisms and pathophysiology of the ARDs, eight of which were on SLE [29–43, 48, 52, 55, 56, 58, 63–67]. Treatment with either PARP1 inhibitors or PARP1 siRNA in nephritis- and RA-based models were reported to elicit the following effects: (i) reducing pro-inflammatory mediator expression levels (including IL-17 and TNF-α); (ii) downregulating mRNA expressions of the cell adhesion molecules ICAM-1 and VCAM-1; (iii) disrupting transcription of various enzymes including iNOS and MMP-2; (iv) hindering reactive oxygen species production; and iv. decreasing inflammatory cell and T-cell subset levels [29, 30, 33, 35, 36, 38]. Consistent with these studies, in a separate paper, arthritis severity was alleviated through suppression of neutrophil infiltration and significant reduction in the inflammatory mediator expression (including pro-inflammatory cytokines IL-1β and MCP-1) in PARP1-deficient mice [41], in arthritic mice treated with a PARP1 inhibitor [34], and in fibroblast-like RA patient synoviocytes treated either with PARP1 inhibitors or PARP1 siRNA [33]. Likewise, PARP1 was shown to be activated during nephritis pathogenesis, with PARP1 wild-type mice displaying high mortality rates, while the absence of PARP1, either through PARP1-knockout mice or pharmacological inhibition of PARP1, led to significantly reduced levels of blood urea nitrogen and proteinuria, decreased thrombotic and necrotic lesions and glomerulosclerosis, attenuated TNF-α levels, slower lymphocyte infiltration, and, ultimately, improved survival rates [35]. At the tissue level, PARP1 expression was upregulated, downregulated or remained unaffected in certain organs across ARDs. For example, PARP1 expression levels remained unaffected in the spleen of mice with SLE or progressive lupus nephritis [64].

b) Protective role of PARP1 in ARD pathogenesis

Some studies demonstrated that PARP1 affects certain genes and inflammatory mediators to confer a protective effect against some ARDs. In one such study, PARP1 repressed the transcription of the anti-inflammatory cytokine IL-10, including the promoter activities of two promoter SNPs GCC and ATA. However, when PARP1 was inhibited with 3-AB, IL-10 promoter activities of GCC and ATA were increased leading to elevated IL-10 [32]. Similarly, AIQ-treated arthritic mice and PARP1-expressing mice administered with 5-AIQ, displayed increased IL-10 levels in their joints [34] and kidneys, respectively [35]. PBMCs from SLE patients, treated with either PJ34 or PARP1 siRNA, had an upregulation in IL-10 production [39]. Treatment with a PARP1 inhibitor or PARP1 siRNA reduced TNF-induced JNK expression and phosphorylation, and impaired AP-1 and NF-κB binding activities [33]. Of note, 5-AIQ and AIQ significantly upregulated Treg cell population by enhancing Foxp3 production, and reducing Th17-expressing cell population and NF-κB p65 expression [30]; while downregulating Th1 autoreactive responses and lessening Th1 cytokine production [34].

c) Interactors of PARP1

PARP1 interacts with NF-κB in RA synovial cells to upregulate promoter activity and receptor tyrosine kinase gene ERBB2 transcription [36]. However, when PARP1 was transiently suppressed with siRNA, ERBB2 expression decreased, implicating that PARP1 regulates both ERBB2 expression and RA synovial cell proliferation [36].

PARP1 was also found to be associated with RA risk polymorphism CCR6BNP in a sequence- and allele-specific manner to regulate CCR6 expression through its PARylation activity [37]. Disrupting PARP1 using an inhibitor, siRNA or shRNA significantly reduced CCR6 expression [37]. On the other hand, in another study, PARP1 had low expression levels in RA, and was one of four hub genes identified from ten key programmed cell death (PCD)-related differentially expressed genes (DEGs) [58]. Furthermore, significant differences in PARP1 expression levels were demonstrated during differentiation of myeloid cell subsets including macrophages [58]. In a separate study focusing on SLE pathogenesis, PARP1 was identified as one of two central hub proteins to link the post-translational modifications with transcriptional alterations within the regulatory protein network [67].

Taken together, these studies demonstrate that PARP1 plays a key role in the pathophysiology of arthritis and nephritis, and its absence elicits protection against these conditions, raising the possibility that PARP1 could be a potential therapeutic target [29, 30, 33–36, 38, 41]. This may be achieved through two strategies: (a) on a molecular and genetic level by reducing pro-inflammatory mediator expression levels and interacting with specific binding partners in the protein network milieu, and (b) on a cellular level by attenuating T-cell subset production and dampening immune cell infiltration.

d) Role of PARP1 in DNA damage response

In other studies, the ability to activate PARP1 to produce PAR chains, respond to and repair the DNA damage following irradiation, was substantially hindered in SLE and SSc patients, implying that PARP1 functionality is defective across these conditions [31, 40, 42, 43, 52]. As a result, PARP1 cleavage was accentuated, PAR polymer synthesis was low, and intracellular NAD^+^ concentration remained high due to NAD^+^ not being used efficiently as a substrate by PARP1 to synthesise PAR and repair DNA damage [31, 42, 43, 52]. Similarly, in lupus-prone mice, PARP1 levels were elevated and DNA damage response pathway was rescued following their treatment with the peroxisome proliferator activator receptor β/δ, GW0742 [56].

PARP1 was also shown to be downregulated in SSc patients compared with the controls due to PARP1 promoter hypermethylation [63]. This was observed along with significantly greater DNA damage levels and reduced expression of base excision repair genes including PARP1 [63].

Concomitantly, in patient and murine SSc fibroblasts, a key role of cytokine TGF-β was observed in suppressing PARP1 expression through PARP1 promoter hypermethylation and recruiting PARP1 to interact with the signal transducer Smad3 to upregulate TGF-β signalling [40]. This subsequently stimulated persistent fibroblast activation and progression of fibrosis, characteristic of SSc [40]. In parallel to these findings, apoptosis in SLE and SSc patients’ PMCs was greater, indicating that the cells undergo apoptosis following failure to successfully repair DNA damage [43]. One study proposed that the impaired PARP activity observed in SLE patients occurs at the transcriptional level [48]. PARP mRNA concentrations were ten-fold lower in lymphocytes from SLE patients compared with controls [48]. No such decrease was observed in the lymphocytes from APLS patients [48]. No significant differences in PARG activity were reported in the included studies, between the patient and control groups, suggesting that PAR chain turnover is not restricted by limiting PARG activity [31].

Overall, these studies reveal how PARP1 activity is disrupted in some ARDs, particularly SLE and SSc, and this subsequently renders a knock-on effect on the downstream pathways. Restoring PARP1 functionality is imperative for ameliorating the pathogenesis in both molecular and epigenetic manners.

### Genetic variants and polymorphisms

Eight papers investigated whether PARP alleles are associated with the risk of developing ARDs especially SLE, APLS and RA [23, 24, 44, 46, 54, 57, 60, 62]. Some observations were consistent, while other studies reported conflicting findings.

Investigations in individuals from Korea, Turkey, the Netherlands and Pakistan revealed that none of the PARP1 polymorphisms were significantly associated with RA susceptibility, disease severity, or with the age of onset of RA [23, 24, 57, 62]. One PARP1 SNP that was examined is + 40329T$\:\to\:$C(Val762Ala), which showed no association with RA risk, suggesting that this allele is redundant as a candidate biomarker gene for screening RA patients and unlikely a genetic risk factor for RA [23, 24]. In contrast, + 40329T$\:\to\:$C(Val762Ala) was shown to be significantly associated with an increased risk of inflammatory arthritis in Korean SLE patients [46]. Another study demonstrated two unique conserved PARP1 haplotypes in a Spanish population, predisposing to RA [60]. These haplotypes were: haplotype A (410T–[A]10–[CA]10–12–1362 C), which includes short PARP1 CA alleles (410T–[A]10–short CA alleles–1362 C); and haplotype B (410 C–[A]11–[CA]13–20–1362T), which has long PARP1 CA variants (410 C–[A]11–long CA alleles–1362T) [60]. Recent studies on another PARP1 polymorphism, Val76Ala, was recently found to be significantly elevated in an RA population in Pakistan, and thus linked to increased RA risk [62].

PARP1 polymorphisms were also not significantly associated with susceptibility to SLE or APLS among French and Korean populations [44, 46]. However, two SNPs, −1963A$\:\to\:$G and + 28077G$\:\to\:$A, were significantly associated with an increased risk of nephritis in Korean SLE patients [46]. However, −1963A$\:\to\:$G rendered a protective effect for inflammatory arthritis in the same population [46]. On the other hand, one study, including individuals of multiple ethnicities, reported frequent transmission of 85-bp allele to offsprings of individuals affected by SLE [54]. This PARP1 polymorphism might be linked to SLE risk within the chromosome 1q41–q42 region, and is likely to confer defective DNA repair and abnormal apoptosis, thereby predisposing to SLE [54]. However, 97-bp SNP was frequently transmitted to the unaffected offspring, thus eliciting a protective role against SLE [54].

Taken together, these reports present several PARP1 polymorphisms, each displaying unique characteristics, that have been identified across multiple ethnicities and populations. As a result, these SNPs could serve as potential markers for examining the risk of specific populations developing these conditions.

### Diagnosis and biomarkers

a) Role of PARP1 detection in SLE

Eleven studies examined the effectiveness of PARP1 as a diagnostic tool and biomarker across several ARDs [45, 47, 49–51, 53, 55, 59, 61, 65, 66]. Most studies detected high PARP1- or PAR-specific autoantibody concentrations in SLE patients, indicating a broad relationship between DNA damage and autoimmune disorders [45, 49–51, 53, 55, 66]. For example, SLE patients’ sera contained high IgG antibody levels, which reacted with PAR and the peptide F2 (corresponding to PARP1 domain involved in recognising DNA breaks) [55]. These autoantibodies are generally characterised by subcellular localisation in the nucleus [66], which is where DNA damage repair primarily takes place in the cells.

Immunoassays involving autoantibodies against PARP-related enzymes or PARP fragments were reported in two papers, examining their ability in detecting SLE clinical activity. An immunoassay involving the thermozyme, PARP*so*, was employed to detect anti-PARP antibodies in SLE patients [45]. In another study, the PARP C-terminal fragment ADPCF was found to have high sensitivity to autoantibodies in SLE patients’ sera [47].

b) Role of PARP1 detection in other ARDs

PARP1 may also be affected as a downstream target from pathways which are impaired in specific ARDs other than SLE. For instance, ERK pathway is impeded in IgA nephropathy and Henoch-Schonlein purpura patients, and PARP1 is one of two proteins to be downregulated as a consequence of this, leading to epigenetic dysregulation [65]. In a separate study, RA patients with periodontal disease had elevated detection levels of PARP1 as a cardiovascular disease risk biomarker, compared with RA patients without periodontal disease [59]. Additionally, another strategy by which PARP1 detection can be studied in ARDs is by analysing its expression and determining to what extent this contributes to genetic ‘signatures’. For instance, it was recently demonstrated that PARP1 was identified via protein-protein interaction analysis as part of a series of hub genes to contribute to the programmed cell death ‘signature’ which indicated programmed cell death resistance or sensitivity following methotrexate treatment in RA FLS [61].

Overall, given that PARP1 participates in key biological processes such as DNA damage response and genome stability maintenance, and is cleaved during apoptosis, these findings implicate that PARP1, and autoantibodies with a high specificity, sensitivity and reactivity against PARP1 or its fragments, are potential biomarkers for screening and diagnosing ARDs, especially SLE, assessing disease progression and analysing PAR metabolism [45, 47, 49–51, 53, 55, 65, 66].

## Discussion

This SLR is the first to comprehensively review the role of PARP1 as a potential diagnostic and therapeutic strategy in ARDs. We highlight the importance of PARP1 in the pathogenesis and treatment of ARDs, both with regards to inflammatory mediators, especially in RA and nephritis, irrespective of whether PARP1 is targeted with an inhibitor, siRNA or shRNA, and also in PAR synthesis and its role in the DNA damage response, notably in SLE and SSc.

Additionally, we present a comparison of PARP alleles and SNPs associated with susceptibility to developing ARDs, predominantly SLE, APLS and RA. Of note, studies focusing on this aspect were conducted predominantly in populations from European and Asian countries, but not in the Americas or Australasia. Variations in reported results across these studies could be due to numerous reasons. First, genetic differences across populations and ethnicities lead to a complex genetic epidemiology that influences susceptibility to SLE, APLS and RA. Second, PARP1 alleles such as + 40329T$\:\to\:$C(Val762Ala), may interact with specific genes that could induce or suppress its ability to predispose individuals in developing certain ARDs. Third, sample sizes across some studies were relatively small [23, 24].

We have also highlighted the utility of PARP1, and domains affiliated with PARP1, as a diagnostic tool in ARD pathophysiology and screening, by assessing PAR metabolism. High concentrations of autoantibodies specific to PARP1 or PAR are present particularly in SLE patient samples [45, 47, 49–51, 53, 55, 66]. Other reports suggest PARP1 could serve as an indirect biomarker of comorbidities in people with ARDs such as cardiovascular disease [59]. While these observations underline an association between DNA damage and autoimmune disorders, other studies have examined the biochemical characteristics in immune cells, and propose a panel of potential biomarkers that could be implemented as part of regular routine monitoring for ARDs. For example, membrane lipidome profiles from human lymphocytes and erythrocytes demonstrate lipid imbalances that are likely to be linked to inflammatory states and certain ARDs [68]. Overall, PARP1 exhibits multiple functions across ARDs, and our results have clinical and research implications for the prospective role of PARP1 in ARD management.

Our findings expand on previous disease-specific studies, including reports evaluating PARP1 polymorphisms in RA, by integrating evidence across multiple ARDs and experimental systems to demonstrate that PARP1 biology spans several domains such as inflammatory signalling, DNA/NAD metabolism, fibrosis and autoantibody responses.

### PARP1 in the diagnosis or management of ARDs

PARP1 is cleaved during apoptosis through a precisely controlled signal transduction cascade to produce cleavage products. However, this cascade is disrupted in ARD patients [69–73]. Observations from previous reports have demonstrated that autoantibodies can detect apoptotic cleavage products as well as non-phagocytosed fragments derived from the nucleosomes [72–74]. Therefore, these findings make PARP1 a potential target of interest for monitoring ARDs by serving as an indirect marker of apoptosis. As a result, examining PARP1 activity could offer an insight into understanding the relationship between apoptosis and autoantibody production.

### The role of other PARP family members in ARDs

The role of PARP1 is the most widely studied in ARDs, among the PARP family. The functionality of other PARPs remains relatively under-explored. Given that the 17 PARP members share a highly conserved C-terminal catalytic domain [75], the mechanism of action of targeting PARP1 using an inhibitor is likely to affect other PARP enzymes. PARP inhibitors (PARPi) DPQ, ANI and nicotinamide target PARP1 but also inhibit PARP2, albeit less potently [33, 38, 52]. Furthermore, small basic inhibitors that target the nicotinamide-binding site, such as 3-AB, have generalised low selectivity across the PARPs [76]. Over the last two decades, novel PARPi have been developed to confer high selectivity and specificity for specific PARP family members. One approach involves creating potent selective inhibitors that interact with the PARPs at sites that are distinct from the catalytic domain – this has been case for the development of the PARP14 inhibitor H10 [77] and PARP5a/5b (tankyrase) inhibitors [78, 79]. More recently, DB008 has been developed, displaying strong selectivity for PARP16 [80], as well as the next-generation inhibitor, thioparib, which renders a pan-PARP effect, eliciting high affinity across several PARPs including PARP1, PARP2 and PARP7 [81]. It should be noted that PARP7 is different from PARP1 and PARP2, since PARP7 is a mono-ADP-ribosyltransferase and is not usually activated upon DNA damage [14, 82]. Nonetheless, such developments pave the way for an expansion in the potential use and role of inhibitors in the clinical setting. Furthermore, PARP members besides PARP1 have the potential to serve as diagnostic biomarkers for monitoring ARD activity. For example, PARP9 promoter hypomethylation was recently demonstrated in RA and SLE patients compared with the controls [83].

### Alternative therapeutic approaches for ARDs

PARP1 inhibitors are used for treating non-ARD conditions, such as cancer. However, their efficacy may be limited through the acquisition of PARPi resistance. To address this, the effectiveness of alternative interventions, such as drug efflux pump inhibitors, should be considered. Drug efflux pumps encompass the multi-drug resistance-1 (MDR-1) encoding ATP-binding cassette transporter, P-glycoprotein, and transport drugs from the intracellular environment to the cell exterior, giving rise to the development of multi-drug resistance [84–86]. Multi-drug resistance is a problem in ARDs, especially in chronic exposure to successive treatments [87], due to factors such as impaired uptake and delivery of the drug and disrupted drug activation [88]. MDR-1 transcription and P-glycoprotein expression also occur upon stimulation of the lymphocytes with cytokines [87]. Previous studies have reported how corticosteroids and disease-modifying anti-rheumatic drugs, are effluxed from P-glycoprotein-overexpressing lymphocytes [88–94]. Numerous drug efflux pump inhibitors that have been investigated in the context of non-ARD conditions are not yet approved for clinical use [95]. Additional strategies for overcoming PARPi resistance include developing next-generation PARPi such as thioparib which have been reported to overcome earlier-generation PARP1 inhibitor resistance [81] and leveraging PROTAC technology [96].

### Strengths and Limitations

As the first SLR to consider the role of PARP1 and PARPs more broadly in ARDs, our review substantially adds to the literature on novel therapeutic strategies and diagnostic tools for people with ARDs, while synthesising the existing evidence. The broad search strategy employed in this SLR ensured capture of all relevant articles. Inclusion of both human and animal studies facilitated a thorough review of existing and potential future applications of the role of PARP1 in ARDs.

However, our SLR has several limitations. Firstly, there was a high degree of heterogeneity with respect to study design, model systems, and outcomes assessed. As described in the Cochrane Handbook [27], variability in study populations, interventions, outcomes, and methodological approaches represents important sources of clinical and methodological heterogeneity that may limit comparability across studies. This methodological diversity also precluded quantitative synthesis. Consequently, a quantitative meta-analysis was not considered appropriate and findings were synthesised narratively. Although the review followed established systematic review principles, formal risk-of-bias tools were not uniformly applicable because of the broad inclusion of mechanistic, translational and preclinical studies. Secondly, much of the evidence was derived from preclinical or mechanistic studies rather than clinical investigations, so conclusions regarding the translational relevance and therapeutic efficacy in people with ARDs are limited and may warrant further investigation. Many experimental studies also employed different dosing strategies and outcome measures, further making it difficult to determine class effects or establish consistent mechanistic pathways. Thirdly, sample sizes in human studies were small and often focused on specific populations or ethnic cohorts, limiting generalisability. Genetic association findings were also inconsistent across populations, reflecting the complex and multifactorial genetic architecture of ARDs. Finally, the topic under study is at risk of publication bias, since positive mechanistic findings are more likely to be published than negative or neutral results.

## Conclusions

In this SLR, we underscore the importance of PARP1 in the pathogenesis and treatment of ARDs; genetic variants and polymorphisms; and as a biomarker of ARD diagnosis and activity. Our results have clinical and research implications for the prospective role of PARP1 in monitoring and managing ARD. However, further investigation is warranted to confirm (a) whether PARP1 can be specifically and effectively targeted across ARDs, with therapeutic benefit; and (b) whether other PARP family members have overlapping roles with PARP1 in inflammation and immunity. Nonetheless, PARP1 demonstrably has a promising potential role in ARD diagnosis, treatment and management.

## Supplementary Information

Below is the link to the electronic supplementary material.

Supplementary Material 1

Supplementary Material 2

## Data Availability

Data available upon reasonable request.

## Declarations

### Disclaimer

No part of this review is copied, published elsewhere (in whole or in part), or generated by AI.
