# Supplementary material for: PARP1 as a novel therapeutic and diagnostic tool in autoimmune rheumatic diseases: a systematic literature review
Source: Rheumatol Int. 2026 Jun 9;46(6):138. doi: 10.1007/s00296-026-06187-0 (PMC13249679; doi:10.1007/s00296-026-06187-0)
Supplement: Supplementary file 1 — Supplementary Material 1 [file 296_2026_6187_MOESM1_ESM.docx]

**Supplementary Table 1: Summary of findings from 37 studies included in SLR.**

PARP1 as a novel therapeutic and diagnostic tool in autoimmune rheumatic diseases: a systematic literature review

Clinical Reviews in Allergy and Immunology

Malini Dey^1^, Mrinalini Dey^2^

1. Autism Research Centre, Department of Psychiatry, University of Cambridge, Douglas House, 18b Trumpington Road, Cambridge, CB2 8AH; ORCID: 0000-0003-2291-7955
2. Centre for Rheumatic Diseases, King’s College London, Weston Education Centre, Cutcombe Road, London SE5 9RJ; ORCID: 0000-0001-6858-4338

Corresponding author:

Dr Malini Dey; [md2138@cam.ac.uk](mailto:md2138@cam.ac.uk)

| **Study (ref)** | **Type of study*** | **Model system(s)** | **Sample size** | **Type of ARD** | **Intervention**** | **Number**  **in intervention** | **Outcome** | **Conclusion** |
| --- | --- | --- | --- | --- | --- | --- | --- | --- |
| Ahmad *et al.*, 2014 (1) | A | Murine AIA | Not specified | AIA | 3-aminobenzamide (3-AB)*** | Not specified | Decreased pro-inflammatory cytokine and adhesion molecule expression levels. | 3-AB exerts significant anti-arthritic effects including suppressing inflammatory cytokines and mediators. |
| Ahmad *et al.*, 2014 (2) | A | Murine AIA | Not specified | AIA | 5-aminoisoquinolinone (5-AIQ)*** | Not specified | Attenuated inflammation and arthritis pathophysiology. Reduced production of T-cell subset and NF-kB p65. | 5-AIQ is a potential potent anti-inflammatory and anti-arthritic agent in RA. |
| Attia *et al*., 2025 (3) | A | Lupus-prone mice | Not specified | SLE | N.A. | Not specified | Following treatment with agonist of peroxisome proliferator activator receptor β/δ, GW0742, PARP1 levels elevated and DNA damage response alleviated. | Conclusion not given specifically for PARP1. |
| Cerboni *et al.*, 2010 (4) | A | SLE and SSc patients, healthy volunteers | 20 | SLE, SSc | N.A. | N.A. | High NAD and low PARP activation in SLE and SSc patient PMCs. | SLE and SSc patients exhibit a suppressed ability to activate PARP, thus revealing defects in their ability to repair DNA damage. |
| Cerboni *et al.*, 2009 (5) | A | SLE and SSc patients, healthy volunteers | 57 | SLE, SSc | N.A. | N.A. | High NAD concentration and low PARP activation in SLE and SSc patient PMCs, in repairing DNA damage following irradiation. | PARP activation in response to UV irradiated-induced damage is reduced in patients affected by ARDs. PARP play a key role in regulating intracellular NAD concentration. |
| Chen *et al.*, 1996 (6) | A | PBM cells used from SLE patients, healthy volunteers | 25 | SLE | Benzamide*** | 25 | Significantly lower PARP activity and PAR polymer accumulation in SLE subjects. | Smaller number of active enzyme molecules are present. PARP enzyme is defective in SLE. |
| Chung *et al.*, 2007 (7) | A | PARP1 KO mice;  murine macrophage and leukemic human T-cell lines; murine and human monocytes and macrophages | Not specified | SLE | 3-aminobenzamide (3-AB)*** | Not specified | Enhanced IL-10 promoter and transcriptional activities. | PARP1 is physiological regulator of IL-10 specifically in response to apoptotic cells. |
| Delrieu *et al.*, 1999 (8) | B | Patients with sporadic or familial SLE, patients with primary APS, and healthy volunteers | 259 | SLE | N.A. | N.A. | No statistically significant differences between controls and SLE group for distributions of PARP alleles, and PARP promoter CA repeat alleles. | PARP alleles do not influence susceptibility to SLE or primary APS in French Caucasians. No skewing present towards particular PARP CA repeat alleles in patients with SLE or primary APS. |
| Emonts *et al.*, 2011 (9) | B | Non-related Caucasian patients who met 1987 ACR criteria for RA and visited rheumatology outpatient clinic of Erasmus MC university hospital, Medical Centre Rijnmond Zuid or St. Franciscus Gasthuis Hospital | 839 | RA | N.A. | 370 | No PARP SNPs are significantly associated with RA susceptibility, disease severity, or with age of onset of RA. | Please refer to Outcome. |
| Fang *et al.*, 2025 (10) | A | Human RA-related circRNA-seq dataset and mRNA-seq dataset, and murine RA single-cell RNA-seq dataset downloaded from Gene expression omnibus database. | 50 | RA | N.A. | N.A. | PARP1 was expressed at low levels in RA. PARP1 was one of four hub genes identified from ten key programmed cell death (PCD)-related differentially expressed genes (DEGs). | Please refer to Outcome. |
| Faraone-Mennella *et al.*, 2009 (11) | C | SLE patients, healthy volunteers | 67 | SLE | N.A. | 67 | Higher anti-PARP activity in ANA positive patients. In SLE, disease and anti-PARP activity increased in active vs inactive disease. Significant correlation between anti-PARP and disease activity. | PARPS*so* can detect anti-PARP antibodies and play role as serological marker of disease activity in SLE. |
| García *et al*., 2006 (12) | A | Mice with CIA lacking PARP1 | Not specified | CIA | N.A. | N.A. | Reduced severity of arthritis, histological features of joint inflammation cartilage damage, and synovial inflammation score | PARP1 plays role in arthritis progression through impaired IL-1β and MCP-1 production in joints; potential experiemental treatment target. |
| García *et al*., 2008 (13) | A | Fibroblast-like synoviocytes from RA patients | 10 | RA | DPQ***, ANI***, and PARP1 siRNA | 10 (RA patient fibroblast-like synoviocytes used) | Significantly reduced: TNF-induced cytokine and chemokine expression, rheumatoid fibroblast response to TNF; RA fibroblast-like synoviocytes; TNF-induced proliferation and TNF-induced transcription of IL6, MCP-1 and MMP-3. | PARP inhibition reduces inflammatory mediator production and proliferation of RA fibroblast-like synoviocytes (in response to TNF); potential therapeutic benefits in RA. |
| Gonzalez-Rey *et al*., 2007 (14) | A | Mice with CIA | N.A. | CIA | 5-aminoisoquinolinone (AIQ)*** | Not specified | PARP1 inhibition decreased severity of experimental arthritis and AIQ inhibited inflammatory response in CIA. | PARP1 is novel therapeutic target for RA treatment and other chronic autoimmune disorders. 5-AIQ deactivates inflammatory response *in* *vivo* at multiple levels. |
| Honarpisheh *et al*., 2016 (15) | A | RNA isolated from human and murine tissues | Not specified | Acute tissue injury, progressive tissue fibrosis and lupus nephritis | N.A. | N.A. | In adult murine tissues, PARP1 highly expressed in testis, thymus and heart. In mice with progressive tissue injury and fibrosis, PARP1 mRNA expression levels significantly reduced in later phase of nephropathy. PARP1 expression not downregulated in spleen during SLE progression. In mice with progressive lupus nephritis, splenic PARP1 mRNA expression remains unchanged. | No specific conclusion on PARP application. |
| Hur *et al*., 2006 (16) | B | Korean SLE patients, and healthy volunteers | 680 | SLE | N.A. | N.A. | 2 SNPs: -1963A -> G and +28077G -> A significantly associated with increased risk of nephritis. 1 non-synonymous variant +40329T -> C(V762A) significantly associated with elevated risk of arthritis, while -1963A -> G SNP protective against arthritis in Korean SLE patients. 2 SNPs: -1963A -> G and +28077G -> A significantly associated with increased risk of lupus glomerulonephritis in SLE. 2 SNPs: -1963A -> G and +40329T -> C(V762A) and two common haplotypes (ht1 and ht3) significantly associated with arthritis in SLE. | PARP polymorphisms not associated with SLE susceptibility; -1963A -> G, +28077G -> A and +40329T -> C(V762A) significantly associated with nephritis and arthritis in Korean SLE patients. |
| Hussain *et al*., 2025 (17) | B | Pakistani RA patients, and healthy volunteers | 1000 | RA | N.A. | N.A. | PARP1 SNP Val76Ala was significantly increased in RA patients compared with controls. | Elevated mutant genotype frequency of PARP1 Val76Ala allele is linked with significantly increased susceptibility to RA, rendering this polymorphism a potential RA diagnostic marker. |
| Jeoung *et al*., 2004 (18) | C | Human sera obtained from patients with SLE, RA, polymyositis/dermatomyositis, SSc, and primary Sjogren syndrome patients, and healthy volunteers | 213 | SLE | N.A. | 213 | ELISA with domains ADPNF or ET-L2 showed low sensitivity and frequency of reactivity in sera of patients with SLE, RA, SSc and polymyositis or dermatomyositis. ELISA with domains ET-L3 or ADPCF revealed high sensitivity, specificity and frequency of reactivity in SLE sera, but low sensitivity and frequency of reactivity in sera of patients with RA, polymyositis or dermatomyositis, SSc and Sjogren syndrome. Presence of autoantibodies to ADPCF showed no correlation with presence of anti-dsDNA. | Autoantibodies against recombinant PARP protein, ADPCF, is an important serologic marker for laboratory testing for SLE diagnosis. Autoimmune response to PARP is important given that PARP is involved in DNA repair, and is cleaved during apoptosis. |
| Jog *et al*., 2009 (19) | A | Nephrotoxic serum-injected mice to induce nephritis, either expressing PARP1 (PARP1^+/+^) or lacking PARP1(PARP1^-/-^) | Not specified | Nephritis | 5-aminoisoquinolinone (AIQ)*** | Not specified | PARP1 inhibition protective against nephritis, dependent on biological sex of mice – severity of nephritis greater in female PARP1^-/-^ mice. Reduced TNF-α and increased IL-10 transcripts were present, suggesting PARP1 activity may protect mice by reducing proinflammatory cytokine milieu. | PARP1 fundamental in pathogenesis of immune-mediated nephritis in males, implying that pathways contributing to nephritis in male and female may differ. PARP inhibition reduces renal tissue damage by limiting necrotic cell death. PARP1 regulates local inflammation in kidney during nephritis, and thus could be a target for therapeutic intervention in nephritis. |
| Kitamura *et al*., 2005 (20) | A | Synovial cells isolated from RA patients and osteoarthritis patients | 3 | RA | PARP1 siRNA and 3-aminobenzamide (3-AB)*** | 3 (RA patient synovial cells used) | PARP1 expression in RA synovial cell samples higher than OA patient-derived synovial cells. In RA synovial cells, PARP1 binds to NF- κB via proximal region of NF-kappaB binding site to activate ERBB2 gene transcription. PARP1 siRNA transfection and PARP1 inhibition in RA synovial cells reduces ErbB2 expression. | PARP1 involved in pathogenesis of RA, with high expression levels in RA cells. PARP1 is involved in ErbB2 expression in concert with NF- κB, which might be associated with RA synovial cell proliferation. PARP1 plays a key role in ErbB2 overexpression in RA synovial cells. |
| Lee *et al*., 1994 (21) | A | Patients with SLE or antiphospholipid syndrome, healthy volunteers | 16 | SLE | N.A. | 16 (Peripheral blood lymphocytes used) | Mean PARP mRNA levels (and thus activity of PARP) were at least 10X lower in peripheral blood lymphocytes from SLE patients. No decrease observed in peripheral blood lymphocytes from APS patients. | Defect in PARP metabolism observed in SLE patients, occurs at level of transcription or mRNA turnover. |
| Lee *et al*., 2012 (22) | B | Korean RA patients, healthy volunteers | 2181 | RA | N.A. | N.A. | PARP1 polymorphisms not significantly associated with RA susceptibility. | Genetic differences among populations or ethnic groups explain complex genetic epidemiology and may affect RA prevalence. Furthermore, lack of association between PARP1 polymorphisms and RA could be due to functional genetic variants that potentially interact with PARP1. |
| Li *et al*., 2016 (23) | A | Human colon cancer cell line HCT116 cells, human monocyte line THP-1, human T-lymphocyte cell line Jurkat cells, and HeLa cells | N.A. | RA | PARP1 shRNA, PARP1 siRNA, and 3-aminobenzamide (3-AB)*** | N.A. | PARP1 associates with CCR6DNP and exhibits sequence and allele specificity. PARP1 regulates CCR6 expression in human HCT116 cells and Jurkat T cells. PARP1 ADP-ribosylation activity regulates CCR6 expression in human cells. Disruption to PARP1 impairs CCR6 expression. | CCR6DNP is a causal variant that controls expression of CCR6. PARP1 directly regulates CCR6 through RA risk polymorphism CCR6DNP. PARP1 associates with CCR6DNP in sequence- and allele-specific manner. PARP1 activity is relevant for CCR6 regulatory function. |
| Lim *et al*., 2002 (24) | C | Human sera obtained from patients with a range of ARDs, and healthy individuals | 291 | SLE,  Sjogren’s syndrome, SSc, polymyositis/myositis, and RA | N.A. | 291 (Sera used) | Autoantibodies to PARP prevalent in SLE. Clones encoding for PARP showed highest frequencies in sera of SLE patients. Autoantibody to PARP was not found in sera of patients with other rheumatic diseases. | High prevalence of PARP in sera of SLE patients gives an insight into mechanism of pathogenesis leading to SLE. Further functional characterisation of PARP autoantibodies needed to determine value as diagnostic markers or to define clinical subsets of SLE. |
| Ling *et al*., 2020 (25) | C | Human sera obtained from patients with SLE and healthy individuals | 1036 | SLE | N.A. | 1036 (Sera used) | PARP1 autoantibodies expressed at higher levels in SLE. Expression level of serum PARP1-IgG higher in active vs inactive disease. Positive rate of serum PARP1-IgG in SLE group was as high as 69.16%, suggesting serum PARP1-IgG is a potential marker of SLE. | Combined autoantibody panels (including PARP1 autoantibodies) show promise for diagnosis of SLE and for differential diagnosis of other major ARDs. |
| Miesel *et al*., 1995 (26) | A | DBA/I x B10A(4R) mice with potassium peroxochromate-induced arthritis | Not specified | Arthritis | Nicotinamide*** | Not specified | PARP1 inhibition reduced arthritis severity by 35%. Acute inflammatory response impeded and differed significantly from controls. Phagocytic generation of reactive oxygen species attenuated. Upon nicotinamide treatment, arthritis index declined linearly with increasing concentrations, suggesting dependence of arthritis progression on PARP activity. | Study results support hypothesis that oxidative stress-induced alterations in cellular signal transduction pathways play a pivotal role in development of arthritis, which can be suppressed by inhibition of PARP. |
| Milillo *et al*., 2018 (27) | A, C | Patients with sporadic and familial IgAN, patients with HSP and healthy volunteers | 21 (number of heathy volunteers not specified) | IgAN and HSP | N.A. | N.A. | PARP1 downregulated in IgAN and HSP patients. Defective ERK1/2 activation in IgAN and HSP could lead to PARP1 downregulation and potential epigenetic dysregulation. | Defective ERK activation leads to PARP1 downregulation, suggesting IgAN could be consequence of a dysregulated epigenetic maintenance. PARP1 is potential biomarker. |
| Muller *et al*., 1994 (28) | A, C | Human sera obtained from patients with various ARDs, and healthy volunteers | 329 | SLE, pSS, sSS, JCA and MCTD | N.A. | 329 (Sera used) | High IgG antibody levels reacting specifically with peptide F2 observed in sera of patients with SLE, pSS and sSS. Minimal reaction displayed in MCTD and JCA patient sera. Antibodies reacting with whole enzyme or peptide F2 occurred independently from antibodies reacting with PAR. | Antibodies reacting with PARP zinc finger F2 (domain which recognises and interacts with DNA breaks) are present in sera of patients with SLE and Sjogren’s syndrome. |
| Negri *et al*., 1990 (29) | C | Human sera obtained from patients with various ARDs and healthy volunteers | 164 | SLE,  RA, SSc, Sjogren’s syndrome, UCTD, CFA and Sarcoidosis | N.A. | Please refer to sample size. | Positive values, and thus very high pADPRP-specific autoantibody concentration were mostly obtained in patients with SLE, CFA and sarcoidosis. | PARP-specific autoantibodies are present in sera of patients affected by various autoimmune and connective tissue diseases. |
| Onaran *et al*., 2009 (30) | B | DNA isolated from peripheral  blood of Turkish RA patients and healthy volunteers | 293 | RA | N.A. | Please refer to sample size. | Distribution of PARP1 genotypes and alleles did not differ significantly in RA vs non-RA. PARP1 V762A polymorphism unlikely to be involved in RA susceptibility, nora genetic risk factor for RA. No statistically significant differences observed on allelic frequency distribution of Val762Ala between RA patients and controls. | Val762Ala polymorphism is not associated with RA development, so may not function as a candidate gene marker for screening RA patients. |
| Pan *et al*., 2024 (31) | A | Lupus-prone and disease-free control mice, primary CD4 T cells purified from peripheral blood obtained from patients with SLE, and healthy volunteers | 38; number of mice not specified | SLE-like autoimmune disease, SLE, systemic autoimmunity and nephritis | Nicotinamide riboside treatment was used on mice and patient samples. | Please refer to sample size. | Nicotinamide riboside treatment suppresses disease in lupus-prone mice and restores NAD^+^-dependent PARP1 activity in CD4 T cells from SLE patients. PARP1 autoPARylation was significantly decreased but PARP1 cleavage significantly higher in CD4 T cells from SLE patients vs controls. CD4 T cells from SLE patients potentially have low PARP1 activity. Moreover, when SLE CD4+ T cells were treated with nicotinamide riboside chloride, this led to a significant increase in PARylated PARP1 and PAR, implying PARP1 activity was enhanced. | Nicotinamide riboside supplementation restores PARP1 enzymatic activity in CD4 T cells from SLE patients. Findings provide rationale to use NR to treat SLE patients. |
| Panezai *et al*., 2022 (32) | C | Human sera obtained from patients with RA and PD, patients with RA without PD, patients with PD only, and healthy volunteers | 118 | Periodontal disease and RA | N.A. | Please refer to sample size. | RA patients with PD had greater detection levels for ~50% measured markers, including PARP1, compared with patients with RA without PD. A strong biological network was identified amongst these proteins. Protein–Protein interaction (PPI) network analysis (separating RA with PD from RA without PD) revealed PARP1 displaying potential interactions. | Periodontal disease augments CVD-related biomarkers, including PARP1, in RA through shared pathological clusters, thereby enhancing metabolic and skeletal disease protein interactions, independent of autoimmune status. |
| Pascual *et al*., 2003 (33) | B | Spanish RA patients and healthy volunteers | 455 | RA | N.A. | N.A. | 2 unique PARP1 haplotypes detected in patients and controls: haplotype A (410T–[A]10–[CA]10–12–1362C which includes short PARP-1 CA alleles) (410T–[A]10–short CA alleles–1362C), and haplotype B (410C–[A]11–[CA]13–20–1362T), always paired with long PARP-1 CA variants (410C–[A]11–long CA alleles–1362T). Haplotype B overrepresented in RA group vs healthy subjects. 97-bp allele in PARP1 CA is an RA-predisposing marker. | 2 unique conserved PARP1 haplotypes in Spanish population exist. |
| Prasad *et al*., 1989 (34) | C | Human sera obtained from SLE patients and healthy volunteers | 16 | SLE | N.A. | Please refer to sample size. | Simple sensitive method was tested successfully for large scale and quick screening of sera samples for suspected anti-PAR antisera. | Assay can be conveniently used for large scale screening of patient sera, especially sera from patients with ARDs for anti-PAR reactivity in both clinic and laboratory. |
| Su *et al*., 2019 (35) | A | Patients with SLE and healthy volunteers | 54 (PBMCs isolated from peripheral blood obtained from participants used) | SLE | PARP1 siRNA and PJ34*** | Please refer to sample size. | PARP1 downregulated in SLE patients. miR-199-3p regulates PARP1 post-transcriptionally in SLE. miR-199-3p inhibition increases PARP1 levels. Significantly upregulated miR-199-3p inversely correlate with PARP1 expression and positively correlate with IL-10 levels in SLE. PARP1 inhibition enhanced IL-10 production in PBMCs. miR-199-3p targets PARP1 to activate ERK1/2 pathway and promote IL-10 production. siPARP1 treatment rescued decrease in IL-10 secretion induced by miR-199-3p inhibition. ERK1/2 phosphorylation (pERK1/2) associated with miR-199-3p and IL-10 expression, but not PARP1. pERK1/2 expression inhibited by PARP1 overexpression, indicating PARP1 might regulate ERK1/2 activation. | miR-199-3p inhibition is a potential therapeutic strategy for SLE patients. Further studies are required to evaluate detailed mechanisms through which decreased PARP1 enhances phosphorylation of ERK1/2 in SLE patients. |
| Tsao *et al*., 1999 (36) | B | Multiplex families comprising two or more SLE patients (to include parents and other siblings). Simplex families recruited (to include patients with SLE, their parents, and other unaffected siblings) | 124 | SLE | N.A. | N.A. | Overall skewing of PARP allele transmission observed in affected offspring; lack of transmission to unaffected offspring. 9 PARP alleles detected in cohort. 85-bp allele of PARP preferentially transmitted to SLE-affected offspring but not to unaffected. 97-bp PARP allele preferentially transmitted to unaffected offspring, thereby rendering protective effect. | 85-bp allele of PARP confers defective DNA repair and abnormal apoptosis, predisposing to SLE. PARP is an SLE susceptibility gene or is in physical proximity to an SLE susceptibility gene. |
| Vlachogiannis *et al*., 2020 (37) | A | SSc patients and healthy volunteers. | 92 (PBMCs derived from participants used) | SSc | N.A. | Please refer to sample size. | DNA damage levels significantly higher in untreated SSc samples. PARP1 expression decreased 4X in SSc patients vs controls. DNA damage levels in SSc PBMCs correlate significantly with corresponding mRNA expression of type I interferon–induced genes. | Defective DDR/R may exert an exacerbated effect on type I interferon pathway activation, and contribute to tissue fibrosis in SSc. |
| Yamanaka *et al*., 1987 (38) | A, C | Human sera obtained from patients with rheumatic complaints and healthy volunteers; HeLa, HEp-2, and human T lymphoblastoid CCRF-CEM cell lines. | 6 (sera); number of healthy volunteers not specified | Not specified – patients had  rheumatic complaints | N.A. | Please refer to sample size. | High IgG autoantibody concentration to PARP protein. Anti-ADPRP antibodies had distinctive immunofluorescent pattern with HEp-2 cells, reacting intensely with nucleoli and metaphase chromosomes, and diffusely with nucleus. | Presence of specific immune response against enzyme associated with various immunodeficiency syndromes. |
| Zhang *et al*., 2018 (39) | A | SSc patients and healthy volunteer skin biopsies; SSc murine models and non-fibrotic control mice. | 43 participants; 3 murine SSc models represented 3 different groups, such that there were minimum of 6 mice per group | SSc | 3-aminobenzamide (3-AB)*** and PJ34*** | Please refer to sample size. | PARP1 expression reduced in SSc patients and murine models. PARP1 promoter hypermethylated in SSc fibroblasts, and TGFβ regulates PARP1 expression in fibrosis. Inhibition of DNA methyltransferases reduced promoter methylation and reactivated PARP1 expression. PARP1 silenced by TGFβ-induced promoter hypermethylation. PARP1 inhibition enhances fibroblast activation and collagen release. | PARP1 is central regulator of skin fibrosis. PARP1 negatively regulates canonical TGFβ signalling in experimental skin fibrosis. PARP1 downregulation in SSc fibroblasts contributes to hyperactive TGFβ signalling and persistent fibroblast activation in SSc. PARP1 disruption fosters canonical TGFβ signalling, stimulates fibroblast activation and exacerbates experimental skin fibrosis; contributes to persistent fibroblast activation and fibrosis progression in SSc. |
| Zhang *et al*., 2025 (40) | A | SLE patients and healthy volunteers. | 1681 (PBMCs derived from patients and volunteers used) | SLE | N.A. | N.A. | From analysing the molecular mechanisms linking nuclear protein alterations to pathogenesis, PARP1 was identified as one of two central hub proteins to bridge post-translational modifications with transcriptional alterations | Conclusion not given specifically for PARP1. |
| Zhang *et al*., 2026 (41) | C | Mice with CIA. | Not specified | RA, CIA | N.A. | N.A. | Anoikis-related gene signatures are disrupted following methotrexate treatment, indicating persistent anoikis resistance. PARP1 was identified as one of four hub genes through protein-protein interaction analysis to contribute to this signature. | Conclusion not given specifically for PARP1. |

*****The letters represent the following: A) Pathogenesis/Molecular mechanisms and Treatment, B) Alleles/Genetic variants/Polymorphisms/SNPs, and C) Diagnosis/Diagnostic tools/Biomarkers

**Intervention/Exposure describes method of targeting PARP1 which includes: named PARP1 inhibitor***, PARP1 siRNA, PARP1 shRNA, and CRISPR (unless something different is specified)

Abbreviations:

ARD = Autoimmune rheumatic disease

AIA = Adjuvant-induced arthritis

CIA = Collagen-induced arthritis

IgAN = Primary IgA nephropathy

HSP = IgA-mediated disorder: Henoch-Schönlein purpura

JCA = Juvenile chronic arthritis

MCTD = Mixed-connective tissue disease

KO = knockout

RA = Rheumatoid arthritis

SLE = Systemic lupus erythematosus

SSc = Systemic sclerosis

pSS = Primary Sjogren’s syndrome

sSS = Secondary Sjogren’s syndrome

UCTD = Undifferentiated connective tissue disease

CFA = Cryptogenic fibrosing alveolitis

Of note, PARP1 has several alternative names including PARP which is stated in several cells.

**References**

1. Ahmad SF, Attia SM, Zoheir KM, Ashour AE, Bakheet SA. Attenuation of the progression of adjuvant-induced arthritis by 3-aminobenzamide treatment. Int Immunopharmacol. 2014;19(1):52–9.

2. Ahmad SF, Zoheir KM, Bakheet SA, Ashour AE, Attia SM. Poly(ADP-ribose) polymerase-1 inhibitor modulates T regulatory and IL-17 cells in the prevention of adjuvant induced arthritis in mice model. Cytokine. 2014;68(2):76–85.

3. Attia MSM, Al-Hamamah MA, Ahmad SF, Nadeem A, Bakheet SA, Ansari MA, et al. The PPAR-β/δ agonist GW0742 alleviates DNA damage and lupus nephritis in an animal model of systemic lupus erythematosus via restoring DNA repair gene expression. Mutat Res Genet Toxicol Environ Mutagen. 2025;906:503881.

4. Cerboni B, Di Stefano A, Micheli V, Morozzi G, Pompucci G, Sestini S. PARP activity and NAD concentration in PMC from patients affected by systemic sclerosis and lupus erythematosus. Nucleosides Nucleotides Nucleic Acids. 2010;29(4-6):471–5.

5. Cerboni B, Morozzi G, Galeazzi M, Bellisai F, Micheli V, Pompucci G, et al. Poly(ADP-ribose) polymerase activity in systemic lupus erythematosus and systemic sclerosis. Hum Immunol. 2009;70(7):487–91.

6. Chen HY, Pertusi RM, Kirkland JB, Rubin BR, Jacobson EL. Biochemical characterization of ADP-ribose polymer metabolism in SLE. Lupus. 1996;5(1):14–21.

7. Chung EY, Liu J, Zhang Y, Ma X. Differential expression in lupus-associated IL-10 promoter single-nucleotide polymorphisms is mediated by poly(ADP-ribose) polymerase-1. Genes Immun. 2007;8(7):577–89.

8. Delrieu O, Michel M, Francès C, Meyer O, Michel C, Wittke F, et al. Poly(ADP-ribose) polymerase alleles in French Caucasians are associated neither with lupus nor with primary antiphospholipid syndrome. Arthritis & Rheumatism. 1999;42(10):2194–7.

9. Emonts M, Hazes MJ, Houwing-Duistermaat JJ, van der Gaast-de Jongh CE, de Vogel L, Han HK, et al. Polymorphisms in genes controlling inflammation and tissue repair in rheumatoid arthritis: a case control study. BMC Med Genet. 2011;12:36.

10. Fang Y, Xu N, Shen J, Chen H, Li G. Exploration of the Regulatory Network of Programmed Cell Death Genes in Rheumatoid Arthritis Based on Blood-Derived circRNA Transcriptome Information and Single-Cell Multi-omics Data. Biochem Genet. 2025;63(6):5361–82.

11. Faraone-Mennella MR, Scarpa R, Petrella A, Manguso F, Peluso R, Farina B. Detecting clinical activity in systemic lupus erythematosus with an archaeal poly(ADP-ribose) polymerase-like thermozyme: a pivotal study. Biomarkers. 2009;14(6):381–7.

12. García S, Bodaño A, González A, Forteza J, Gómez-Reino JJ, Conde C. Partial protection against collagen antibody-induced arthritis in PARP-1 deficient mice. Arthritis Res Ther. 2006;8(1):R14.

13. García S, Bodaño A, Pablos JL, Gómez-Reino JJ, Conde C. Poly(ADP-ribose) polymerase inhibition reduces tumor necrosis factor-induced inflammatory response in rheumatoid synovial fibroblasts. Ann Rheum Dis. 2008;67(5):631–7.

14. Gonzalez-Rey E, Martínez-Romero R, O'Valle F, Aguilar-Quesada R, Conde C, Delgado M, et al. Therapeutic effect of a poly(ADP-ribose) polymerase-1 inhibitor on experimental arthritis by downregulating inflammation and Th1 response. PLoS One. 2007;2(10):e1071.

15. Honarpisheh M, Desai J, Marschner JA, Weidenbusch M, Lech M, Vielhauer V, et al. Regulated necrosis-related molecule mRNA expression in humans and mice and in murine acute tissue injury and systemic autoimmunity leading to progressive organ damage, and progressive fibrosis. Biosci Rep. 2016;36(6).

16. Hur JW, Sung YK, Shin HD, Park BL, Cheong HS, Bae SC. Poly(ADP-ribose) polymerase (PARP) polymorphisms associated with nephritis and arthritis in systemic lupus erythematosus. Rheumatology (Oxford). 2006;45(6):711–7.

17. Hussain MZ, Khan MH, Haris MS, Huzaira R, Munawar A, Haq MFU, et al. Association of DNA damage response pathway genes with rheumatoid arthritis risks: a case-control study. Sci Rep. 2025;15(1):20937.

18. Jeoung D, Lim Y, Lee EB, Lee S, Kim HY, Lee H, et al. Identification of autoantibody against poly (ADP-ribose) polymerase (PARP) fragment as a serological marker in systemic lupus erythematosus. J Autoimmun. 2004;22(1):87–94.

19. Jog NR, Dinnall JA, Gallucci S, Madaio MP, Caricchio R. Poly(ADP-ribose) polymerase-1 regulates the progression of autoimmune nephritis in males by inducing necrotic cell death and modulating inflammation. J Immunol. 2009;182(11):7297–306.

20. Kitamura T, Sekimata M, Kikuchi S, Homma Y. Involvement of poly(ADP-ribose) polymerase 1 in ERBB2 expression in rheumatoid synovial cells. Am J Physiol Cell Physiol. 2005;289(1):C82–8.

21. Lee JS, Haug BL, Sibley JT. Decreased mRNA levels coding for poly(ADP-ribose) polymerase in lymphocytes of patients with SLE. Lupus. 1994;3(2):113–6.

22. Lee KA, Bang SY, Park BL, Kim JH, Shin HD, Bae SC. Lack of association between poly(ADP-ribose) polymerase (PARP) polymorphisms and rheumatoid arthritis in a Korean population. Rheumatol Int. 2012;32(1):91–6.

23. Li G, Cunin P, Wu D, Diogo D, Yang Y, Okada Y, et al. The Rheumatoid Arthritis Risk Variant CCR6DNP Regulates CCR6 via PARP-1. PLoS Genet. 2016;12(9):e1006292.

24. Lim Y, Lee DY, Lee S, Park SY, Kim J, Cho B, et al. Identification of autoantibodies associated with systemic lupus erythematosus. Biochem Biophys Res Commun. 2002;295(1):119–24.

25. Ling HZ, Xu SZ, Leng RX, Wu J, Pan HF, Fan YG, et al. Discovery of new serum biomarker panels for systemic lupus erythematosus diagnosis. Rheumatology (Oxford). 2020;59(6):1416–25.

26. Miesel R, Kurpisz M, Kröger H. Modulation of inflammatory arthritis by inhibition of poly(ADP ribose) polymerase. Inflammation. 1995;19(3):379–87.

27. Milillo A, Molinario C, Costanzi S, Vischini G, La Carpia F, La Greca F, et al. Defective activation of the MAPK/ERK pathway, leading to PARP1 and DNMT1 dysregulation, is a common defect in IgA nephropathy and Henoch-Schönlein purpura. J Nephrol. 2018;31(5):731–41.

28. Muller S, Briand JP, Barakat S, Lagueux J, Poirier GG, De Murcia G, et al. Autoantibodies reacting with poly(ADP-ribose) and with a zinc-finger functional domain of poly(ADP-ribose) polymerase involved in the recognition of damaged DNA. Clin Immunol Immunopathol. 1994;73(2):187–96.

29. Negri C, Scovassi AI, Cerino A, Negroni M, Borzì RM, Meliconi R, et al. Autoantibodies to poly(ADP-ribose)polymerase in autoimmune diseases. Autoimmunity. 1990;6(3):203–9.

30. Onaran I, Tezcan G, Ozgönenel L, Cetin E, Ozdemir AT, Kanigür-Sultuybek G. The Val762Ala polymorphism in the poly(ADP-ribose) polymerase-1 gene is not associated with susceptibility in Turkish rheumatoid arthritis patients. Rheumatol Int. 2009;29(7):797–800.

31. Pan W, Tsokos MG, Scherlinger M, Li W, Tsokos GC. The PP2A regulatory subunit PPP2R2A controls NAD(+) biosynthesis to regulate T cell subset differentiation in systemic autoimmunity. Cell Rep. 2024;43(7):114379.

32. Panezai J, Ghaffar A, Altamash M, Åberg M, Van Dyke TE, Larsson A, et al. Periodontal Disease Augments Cardiovascular Disease Risk Biomarkers in Rheumatoid Arthritis. Biomedicines. 2022;10(3).

33. Pascual M, López-Nevot MA, Cáliz R, Ferrer MA, Balsa A, Pascual-Salcedo D, et al. A poly(ADP-ribose) polymerase haplotype spanning the promoter region confers susceptibility to rheumatoid arthritis. Arthritis Rheum. 2003;48(3):638–41.

34. Prasad S, Thraves P, Kanai Y, Smulson M, Dritschilo A. A dot-blot method for screening polyclonal and monoclonal antisera to poly(ADP-ribose). J Immunol Methods. 1989;116(1):79–85.

35. Su X, Ye L, Chen X, Zhang H, Zhou Y, Ding X, et al. MiR-199-3p promotes ERK-mediated IL-10 production by targeting poly (ADP-ribose) Polymerase-1 in patients with systemic lupus erythematosus. Chem Biol Interact. 2019;306:110–6.

36. Tsao BP, Cantor RM, Grossman JM, Shen N, Teophilov NT, Wallace DJ, et al. PARP alleles within the linked chromosomal region are associated with systemic lupus erythematosus. J Clin Invest. 1999;103(8):1135–40.

37. Vlachogiannis NI, Pappa M, Ntouros PA, Nezos A, Mavragani CP, Souliotis VL, et al. Association Between DNA Damage Response, Fibrosis and Type I Interferon Signature in Systemic Sclerosis. Front Immunol. 2020;11:582401.

38. Yamanaka H, Willis EH, Penning CA, Peebles CL, Tan EM, Carson DA. Human autoantibodies to poly(adenosine diphosphate-ribose) polymerase. J Clin Invest. 1987;80(3):900–4.

39. Zhang Y, Pötter S, Chen CW, Liang R, Gelse K, Ludolph I, et al. Poly(ADP-ribose) polymerase-1 regulates fibroblast activation in systemic sclerosis. Ann Rheum Dis. 2018;77(5):744–51.

40. Zhang Q, Xia Y, Li X, Li J, Tang D, Dai Y, et al. Molecular Signatures of Nuclear Protein Alterations in Systemic Lupus Erythematosus across Disease Stages. J Proteome Res. 2025;24(11):5780–92.

41. Zhang Y, Cai B, Shao J, Chen N, Zhu Y, Bao W, et al. The anoikis signature in rheumatoid arthritis: Insights into methotrexate resistance and the complementary therapeutic role of triptolide. Eur J Pharmacol. 2026;1015:178547.
