# Supplementary material for: PARP1 as a novel therapeutic and diagnostic tool in autoimmune rheumatic diseases: a systematic literature review
Source: Rheumatol Int. 2026 Jun 9;46(6):138. doi: 10.1007/s00296-026-06187-0 (PMC13249679; doi:10.1007/s00296-026-06187-0)
Supplement: Supplementary file 2 — Supplementary Material 2 [file 296_2026_6187_MOESM2_ESM.docx]

**Supplementary File 1**

1. **Search strategy in Medline and Embase**

"Autoimmune Rheumatic Disease" or "Autoinflammatory diseases" or "AIRD" or "Rheumatic Disease" or "Rheumatic musculoskeletal diseases" or "Inflammatory Arthritis" or "inflammatory polyarthropathy" or "Spondyloarth*" or "Rheumatoid*" or "Psoriatic Arthritis" or "Ankylosing Spondylitis" or "Axial Spondyoarth*" or "Seronegative inflam*" or "LUPUS" or "systemic lupus erythematosus" or "Sjogre*" or "systemic sclerosis" or "Cutaneous sclerosis" or "limited sclerosis" or "cutaneous limited sclerosis" or "mixed connective tissue disease" or "connective tissue disease" or "undifferentiated connective tissue disease" or "CTD" or "limited scleroderma" or "Scleroderma" or "antiphospholipid syndrome" or "inflammatory myopathy" or "myositis" or "anti-synthetase syndrome" or "undifferentiated connective tissue disease" or "dermatomyositis" or "Vasculitis" or "Kawasaki disease" or "Takayasu arteritis" or "Polyarteritis nodosa" or "PAN" or "giant cell arteritis" or "GCA" or "Temporal arteritis" or "Eosinophilic granulomatosis” or “Juvenile-Onset Lupus” or “Juvenile dermatomyositis” or “Juvenile myositis” or “Juvenile scleroderma” or “Juvenile Idiopathic Arthritis” or “JIA” or “Juvenile Arthritis” or “Oligoarticular juvenile arthritis” or “Polyarticular juvenile arthritis” or “Systemic juvenile arthritis” or “Enthesitis-related juvenile arthritis” or “Psoriatic juvenile arthritis” or “Henoch-Schönlein purpura”

AND

“poly(ADP-ribose) polymerase*” or “PARP*” or “ADP-ribosyl transferase *Diphtheria* toxin-like” or “ARTD*” or “Poly-poly(ADP-ribose) polymerase” or “Poly-PARP” or “poly(ADP-ribose) synthase” or “PARS” or “ADP-ribosyltransferase” or “ADP-ribosyl transferase” or “ADPRT” or “VaultPARP” or “vPARP” or “Tankyrase*” or “TNK*” or “2,3,7,8-Tetrachlorodibenzo-p-dioxin (TCDD)-induced poly-adenosine diphosphate (ADP)-polymerase” or “TIPARP” or “tiPARP” or “B-aggressive lymphoma*” “BAL*” or “ZC3HDC1” or “ZC3H1” or “Zinc Finger CCCH Domain-Containing Protein 1” or “Zinc Finger CCCH-Type Domain Containing 1” or “Zinc Finger CCCH Type Domain Containing 1” or “zinc finger antiviral protein” or “ZAP” or “zinc finger CCCH-type and antiviral 1” or “zinc finger CCCH-type containing, antiviral 1” or “ZC3HAV1” or “COAST6” or “CoaSt6” or “collaborator of Stat6” or “poly(ADP-ribose) glycohydrolase*” or “PARG*” or “Olaparib” or “Lynparza” or “AZD-2281” or “MK-7339” or “KU0059436” or “Veliparib” or “ABT-888” or “NSC-737664” or “Talazoparib” or “Talzenna” or “BMN 673” or “BMN-673” or “Rucaparib” or “Rubraca” or “AG-014699” or “AG-14699” or “CO-338” or “PF-01367338” or “PF-1367338” or “Niraparib” or “Zejula” or “MK-4827” or “Saruparib” or “AZD5305” or “AZD-5305” or “AZD-530” or “2589531-76-8” or “16MZ1V3RBT” or “PJ34” or “PJ34 hydrochloride” or “PJ-34 hydrochloride hydrate” or “MFCD22581510” or “CAS 344458-15-7” or “Pamiparib” or “BeiGene-290” or “BGB-290” or “BGB-290-BeiGene” or “PARTRUVIX” or “DPQ” or “3,4-Dihydro-5[4-(1-piperindinyl)butoxy]-1(2H)-isoquinoline” or “ITK7” or “AZ9482” or “2-(4-(3-((4-Oxo-3,4-dihydrophthalazin-1-yl)methyl)benzoyl)piperazin-1-yl)nicotinonitrile” or “EB-47 dihydrochloride” or “EB-47” or “BGP-15” or “BGP 15” or “BGP15” or “Senaparib” or “1,5-Isoquinolinediol” or “IQD” or “1,5-Dihydroxyisoquinoline” or “5-Hydroxy-1(2H)-isoquinoline or 5-hydroxy-1(2H)-isoquinolinone” or “1,5-Dihydroxyisoquinoline” or “NSC 65585” or “NU1025” or “8-Hydroxy-2-methylquinazoline-4-one” or “Basroparib” or “STP1002”

1. **Search strategy in Directory of Open Access Journals**

poly(ADP-ribose) polymerase OR PARP

1. **Search strategy in Scopus**

TITLE-ABS-KEY ( ( "autoimmune rheumatic disease" OR "inflammatory arthritis" OR rheumatoid* OR "psoriatic arthritis" OR "ankylosing spondylitis" OR spondyloarth* OR lupus OR "systemic lupus erythematosus" OR sjogren* OR "systemic sclerosis" OR scleroderma OR "connective tissue disease" OR "mixed connective tissue disease" OR vasculitis OR myositis OR dermatomyositis OR "juvenile idiopathic arthritis" OR JIA OR "antiphospholipid syndrome" ) ) AND TITLE-ABS-KEY ( ( PARP* OR "poly(ADP-ribose) polymerase*" OR PARG* OR "poly(ADP-ribose) glycohydrolase*" OR tankyrase* OR olaparib OR niraparib OR rucaparib OR talazoparib OR veliparib OR pamiparib OR senaparib OR saruparib OR PJ34 OR DPQ OR NU1025 OR BGP-15 ) )

1. **Search strategy in Web of Science**

TS=("autoimmune rheumatic disease" OR "inflammatory arthritis" OR rheumatoid* OR "psoriatic arthritis" OR "ankylosing spondylitis" OR spondyloarth* OR lupus OR "systemic lupus erythematosus" OR sjogren* OR "systemic sclerosis" OR scleroderma OR "connective tissue disease" OR "mixed connective tissue disease" OR vasculitis OR myositis OR dermatomyositis OR "juvenile idiopathic arthritis" OR JIA OR "antiphospholipid syndrome" ) AND TS=( PARP* OR "poly(ADP-ribose) polymerase*" OR PARG* OR "poly(ADP-ribose) glycohydrolase*" OR tankyrase* OR olaparib OR niraparib OR rucaparib OR talazoparib OR veliparib OR pamiparib OR senaparib OR saruparib OR PJ34 OR DPQ OR NU1025 OR BGP-15)
